# Comparative rates of evolution in endosymbiotic nuclear genomes

**DOI:** 10.1186/1471-2148-6-46

**Published:** 2006-06-14

**Authors:** Nicola J Patron, Matthew B Rogers, Patrick J Keeling

**Affiliations:** 1 Canadian Institute for Advanced Research, Department of Botany, University of British Columbia, Vancouver, V6T 1Z4, Canada

## Abstract

**Background:**

The nucleomorphs associated with secondary plastids of cryptomonads and chlorarachniophytes are the sole examples of organelles with eukaryotic nuclear genomes. Although not as widespread as their prokaryotic equivalents in mitochondria and plastids, nucleomorph genomes share similarities in terms of reduction and compaction. They also differ in several aspects, not least in that they encode proteins that target to the plastid, and so function in a different compartment from that in which they are encoded.

**Results:**

Here, we test whether the phylogenetically distinct nucleomorph genomes of the cryptomonad, *Guillardia theta*, and the chlorarachniophyte, *Bigelowiella natans*, have experienced similar evolutionary pressures during their transformation to reduced organelles. We compared the evolutionary rates of genes from nuclear, nucleomorph, and plastid genomes, all of which encode proteins that function in the same cellular compartment, the plastid, and are thus subject to similar selection pressures. Furthermore, we investigated the divergence of nucleomorphs within cryptomonads by comparing *G. theta *and *Rhodomonas salina*.

**Conclusion:**

Chlorarachniophyte nucleomorph genes have accumulated errors at a faster rate than other genomes within the same cell, regardless of the compartment where the gene product functions. In contrast, most nucleomorph genes in cryptomonads have evolved faster than genes in other genomes on average, but genes for plastid-targeted proteins are not overly divergent, and it appears that cryptomonad nucleomorphs are not presently evolving rapidly and have therefore stabilized. Overall, these analyses suggest that the forces at work in the two lineages are different, despite the similarities between the structures of their genomes.

## Background

**Figure 1:**
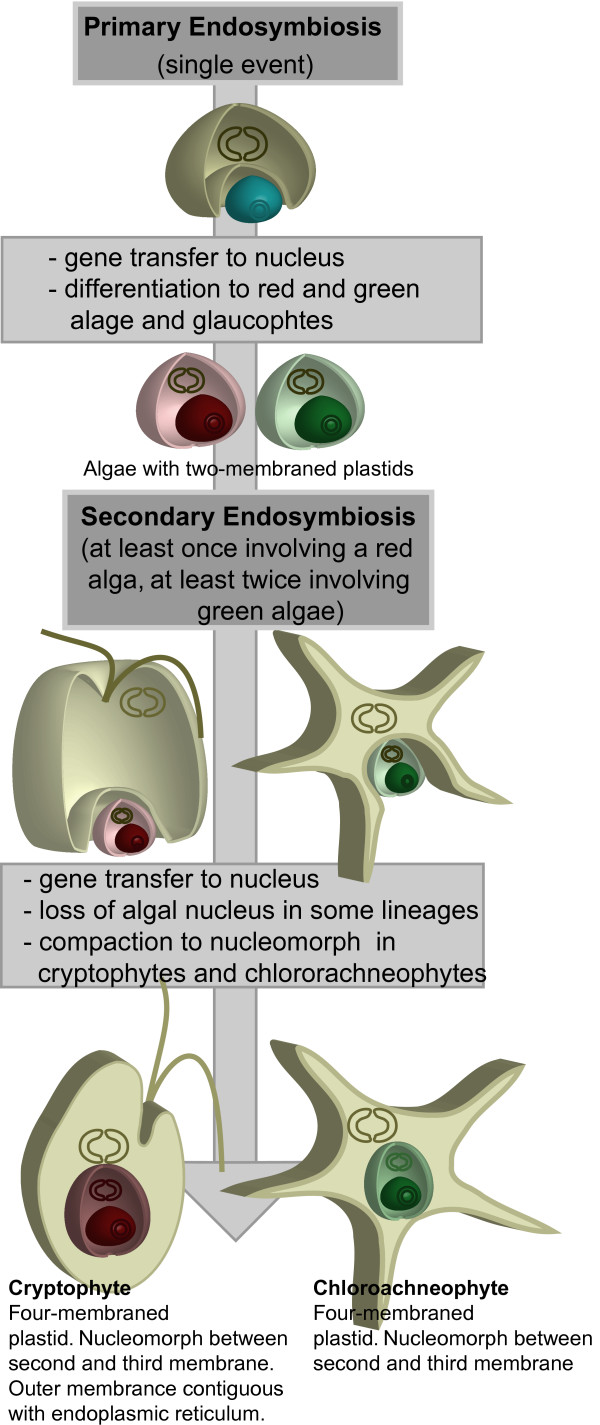
Endosymbiotic events that gave rise to cryptomonads and chlorarachniophytes.

While the primary acquisition of the plastid from a free-living cyanobacterium is believed to have occurred only once [1], plastids have continued to spread through eukaryotes by means of secondary and tertiary endosymbiosis. This is the process whereby a plastid-containing, free-living eukaryote is consumed by another eukaryotic cell and becomes an organelle itself. Primary plastids (exemplified by those of plants) have two membranes, while secondary plastids have additional membranes corresponding to the outer membrane of the engulfed eukaryote and the phageosomal membrane of the host, as well as the original membranes of the primary plastid [2,3], although in some lineages membranes have subsequently been lost. The nucleus of the engulfed cell is, in all but two described cases, absent, and the genes encoding plastid-targeted proteins having been relocated to the host nucleus [4-6]. The exceptions are the cryptomonads and chlorarachniophytes, which contain nucleomorphs, the remnant nuclei of the plastid-containing algae that were engulfed in the secondary endosymbioses that gave rise to these lineages (Figure 1). The cryptomonad endosymbiont is derived from a red alga, while that of chlorarachniophytes is derived from a green alga. Their genomes encode very few genes, and most of them are housekeeping genes for replication, transcription and protein folding and degradation [7,8]. A handful of proteins related to plastid function have also been retained, however, they are relatively few [7,9,10]. The periplastidial space (equivalent to the cytosol of the engulfed alga) itself has specific metabolic processes, such as starch synthesis in cryptomonads, but most of the proteins for these pathways are missing from the nucleomorph genome [7] and are anticipated to be found in the nuclear genome, as has been shown for a few examples [11].

The nucleomorph is often thought of as an anomaly, a rare occurrence, since it is known only in cryptomonads and chlorarachniophytes, but if one considers 'loss or gain' rather than 'presence or absence' then it is perhaps not so anomalous. All lineages that are known to contain secondary plastids (haptophytes, heterokonts, cryptomonads, dinoflagellates, apicomplexans, euglenids and chlorarachniophytes) have ancestors that contained a nucleomorph. Depending on the number of secondary endosymbiotic events that took place, which is still contentious [3,12-14], the number of nucleomorph losses and gains differs. The balance of molecular evidence points to two events involving green algae [15,16] and one involving a red alga [17-19]. With respect to green algae this means one lineage lost its nucleomorph and one retained it. With respect to red algae, this means a single nucleomorph gain (if one accepts the chromalveolate hypothesis [20]) and perhaps only one loss, if cryptomonads are the deepest branch of chromalveolates, or perhaps two if they diverged later. Overall, lineages retaining nucleomorphs may be as common as lineages that lost them, or at least the proportions are very similar. Whatever the case, nucleomorphs existed in the common ancestors of a great deal of algal diversity, so the study of the lineages in which they remain may help us understand the process of secondary (and higher order) endosymbiotic events, especially the reduction and subsequent loss of the enslaved genome.

Cryptomonads and chlorarachniophytes arose from separate endosymbiotic events, and neither host cell nor endosymbiont are very closely related. Yet the nucleomorph genomes of the cryptomonad, *Guillardia theta *[7] and the chlorarachniophyte, *Bigelowiella natans *[8-10] share several characteristics. Both nucleomorph genomes have undergone substantial gene loss and are ultra-compact compared to their free-living relatives in the red and green algae. Some of these features, such as overlapping genes, short intergenic regions, a reduction in elements like transposons, and the presence of multigene transcripts have been found in other compact eukaryotic genomes such as microsporidia [21,22]. Compact genomes and many of these features are common to endosymbionts in general, however, until the sequences of the *G. theta *and *B. natans*, nucleomorph genomes were completed, all known endosymbiont genomes have been of prokaryotic origin. The best examples of prokaryotic endosymbiont genomes are those of the mitochondrion, once a free-living alpha-proteobacterium, and the chloroplast, once a free-living cyanobacterium [1]. Also well described, although not organellar, are the bacterial endosymbionts of insects, of these there are several complete genomes; for example, *Wolbachia *[23-25], *Buchnera *[26], *Wigglesworthia *[27] and *Blochmania *[28], the features of which have been compared and defined [29-31]. These bacteria reside within a range of diverse insects but, while they retain certain distinct genes that can be linked to the physiology of their host, they show similar patterns of genome reduction, strong mutational AT bias and strict amino acid bias at high expression genes [32]; an effect of selection against mutation driven amino acid changes [31,33]. The AT mutational pressure in endosymbionts, is sometimes very extreme; estimated to be a remarkable 90% GC->AT in *Buchnera *[34]. A universal AT mutational bias, has been suggested because many types of spontaneous mutations (e.g. the deamination of cytosine) cause GC to AT changes [35]. The effects of this mutational bias may be more pronounced and gene loss more rapid in small, endosymbiont genomes because they are deficient in at least one DNA repair mechanism, experience strong genetic drift and have experienced a relaxation of selection in the intracellular environment in comparison to free-living existence [31,33].

There is less chromosomal information for eukaryotic obligate intracellular parasites, however certain alveolate and microsporidian genomes show some similar characteristics such as genome compaction [22], AT bias [7,36,37], codon bias [38,39] and extreme divergence. A summary of the features of organelle-, obligate-intracellular- and nucleomorph-genomes is given in Table 1. These features are important to consider as measure of how unusual, or not, nucleomorph genomes are.

**Table 1:** Features of endosymbiont and organelle genomes. '*' – no genome of free-living relative, '?' – not determined.

**Organism**		**Genome Compacted **(compared to free-living relative)	**AT bias**	**Codon Bias**	**Expression bias **(highly expressed genes are less divergent and GC rich)	**Divergent **(compared to free-living relative)
**Organelle genomes of prokaryotic origin**	Mitochondria	Y	Y	Y	Y	Y
	Plastids	Y	Y	Y	Y	Y

**Prokaryotic obligate intracellular symbionts**	*Wolbachia*	Y	Y	Y	Y	Y
	*Wigglesworthia*	Y	Y	Y	Y	Y
	*Buchnera*	Y	Y	Y	Y	Y

**Organelle genomes of eukaryotic origin**	*Guillardia theta *nm	Y	Y	?	?	Y
	*Bigelowiella natans *nm	Y	Y	?	?	Y

**Eukaryotic obligate Intracellular parasites**	Plasmodium	-*	Y	Y	Y	-*
	*Toxoplasma*	-*	Y	?	?	-*
	*Cryptosporidium*	-*	No	?	?	-*
	*E. cuniculi*	Y	No	?	?	Y

With the recent availability of red algal [40] and green algal [41] genomic data we are for the first time in a position to do comparative genomics between nucleomorphs of both cryptomonads and chlorarachniophytes and examples of their free-living relatives, with the plant *Arabidopsis thaliana *serving as an outgroup. Here we test whether the phylogenetically distinct nucleomorph genomes of *G. theta *and *B. natans *have experienced similar evolutionary pressures that influenced genome-wide variation in predictable ways and with the same severity and whether these effects are in common to those described in other enslaved nuclei. Proteins from both nucleomorph genomes have been observed to reside on long branches of phylogenetic trees indicating that they are poorly conserved [42-45], however this has never been investigated at the genomic level. It is also assumed that nucleomorph genes are highly derived because the proteins function within a sub-cellular compartment, the periplastidial space, where selection is relaxed due to reduced interactions with other proteins. However, both the *G. theta *and *B. natans *nucleomorphs encode proteins that are directed to the plastid. Proteins that function in the plastid are presumably subject to similar selection pressures in organisms with nucleomorphs as they are in other algae. We have therefore used plastid proteins encoded in the plastid genome, the nucleomorph, or the nucleus, to examine differences in rates of evolution in the different genomes to determine whether the nucleomorph is evolving at a dissimilar rate to the plastid and nuclear genomes. We also investigate the overall variability of evolutionary rates of nucleomorph-encoded proteins and their homologues in other species to determine if the proteins still encoded within these genomes are generally well conserved, and whether this can shed light on their retention in the nucleomorph. By comparing proteins from the nucleomorph of two cryptomonads, *G. theta *and *Rhodomonas salina*, we also investigate whether cryptomonad nucleomorph genomes are diverging at the same rate as their nuclear genomes.

## Results and discussion

### Plastid-encoded proteins are less divergent than nuclear-encoded plastid-targeted proteins

The plastids of both *G. theta *and *B. natans *use proteins encoded in the nuclear genome, the nucleomorph genome and the plastid itself. Of the 147 proteins encoded in the *G. theta *plastid genome [46] 45 are also present in the plastid genomes of the red alga *C. merolae *and the green plant *A. thaliana*. Of the 57 proteins encoded in the *B. natans *plastid genome, 53 are also present in the plastid genome of the green alga *C. reinhardtii *and *A. thaliana*. One of these proteins, YCF1 proved to be unalignable and was excluded from the analysis. Since the genomes of all plastids are descendents of the cyanobacterial primary plastid ancestor, these proteins are homologues (although some gene duplications have occurred in certain plastid lineages).

**Figure 2:**
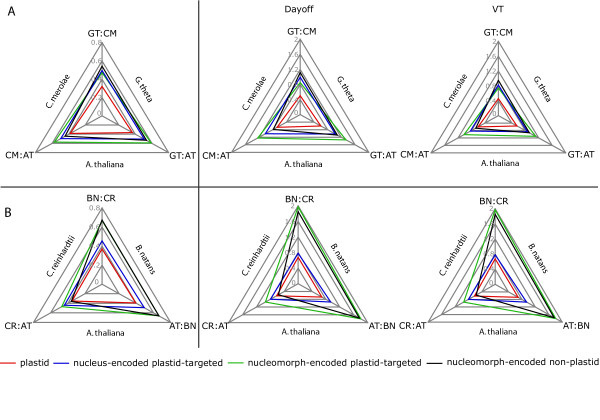
Radar graphs of average distance of plastid-, nucleus-, and nucleomorph-encoded plastid proteins, and nucleomorph-encoded non-plastid proteins of: (A) the cryptophyte *G. theta *(GT), the red algae *C. merolae *(CM) and the plant *A. thaliana *(AT); and (B) the chlororachniophyte *B. natans *(BN), the green algae *C. reinhartii *(CR) and *A. thaliana *(AT) without and with substitution matrices (Dayhoff, VT). In each case the secondary endosymbiont-containing organism is compared to a free living example of its symbiont (red or green algae for A and B, respectively) and the plant *A. thaliana *as an outgroup. Note, scale is different for graphs either without or with substitution matrices.

The average distances, calculated by all methods (with or without substitution matrices, see methods) between the plastid-encoded proteins of *G. theta*, *C. merolae *and *A. thaliana *are smaller than the average distances between the nuclear-encoded proteins (Figure 2a). The distance from *G. theta *to *A. thaliana *and the distance from *C. merolae *to *A. thaliana *is slightly greater than between the *G. theta *and *C. merolae*, indicating that red and green plastids are more distant than primary and secondary red plastids (red, Figure 2a), however the difference is not substantial. The average distances between plastid-encoded proteins of *B. natans*, *A. thaliana *and *C. reinhardtii*, the plastids of which are all of the green lineage, are also smaller than nucleus-encoded proteins (Figure 2b). However, the three plastids are roughly equidistant indicating that secondary endosymbiosis did not affect the speed of divergence of plastid genes in *B. natans *(red, Figure 2b).

Nuclear-encoded plastid genes have been transferred from the plastid genome during endosymbiosis resulting in reduced organelle genomes. Nucleus-encoded plastid-targeted proteins of *G. theta *and *B. natans *[16,47] were identified from ongoing expressed sequence tag (EST) sequencing projects (see methods) by similarity to known plastid proteins and, where present, the characteristics of targeting N-terminal presequences that direct these proteins to their secondary plastid; a signal peptide flowed by a transit peptide [48]. In *G. theta *transit peptides have the characteristics of red algal transit peptides [49], and in *B. natans*, of green algal transit peptides [47]. For each of these proteins homologues were identified from the nuclear genomes of *A. thaliana *and from the nuclear genomes of *C. merolae *(for *G. theta*) or *C. reinhardtii *(for *B. natans*). Twenty-four nucleus-encoded plastid-targeted proteins were found in EST data from *G. theta *for which there were identifiable homologues in *C. merolae *and *A. thaliana*, and 45 plastid proteins were identified from *B. natans *for which there were identifiable homologues from *C. reinhardtii *and *A. thaliana*. A *G. theta *gene encoding an isoform of glycogen (starch) synthase was excluded from the analysis since starch is accumulated in the periplastidial space in this species, but its homologue in *C. merolae *is active in the cytosol and the its homologues in green algae and plants are active in the plastid [50]. Also excluded from the analysis was a nuclear copy of the *tha*4 gene also found in the *G. theta *nucleomorph. The protein encoded by this gene was longer than the nucleomorph protein, which, in comparison to isoforms from other species appears truncated. It is possible that the nuclear *tha4 *gene is a recent transfer that has assumed the function of the nucleomorph-encoded protein and that the truncated, nucleomorph copy is in the process of being lost.

The average distances between nuclear-encoded plastid-targeted proteins of *G. theta*, *A. thaliana *and *C. merolae *are larger than the plastid-encoded proteins, and are almost identical between the three species (blue, Figure 2a). Similarly the average distances between nuclear-encoded plastid-targeted proteins of *B. natans, A. thaliana *and *C. reinhardtii *arealmost equal (blue, Figure 2b), but larger than distances for plastid-encoded proteins from the same taxa. Distances calculated for both plastid and nuclear-encoded proteins using the Dayhoff and VT substitution matrices were larger than the average number of substitutions (i.e. calculated without substitution matrix), which shows that amino acids were most often substituted with similar residues, suggesting functional conservation.

Overall, these analyses show that nucleus-encoded plastid-targeted proteins are on average more divergent than proteins encoded in the plastid genome. Two possible causes for this observation are 1) the rates of general substitution are higher in nuclear genomes, or 2) the genes retained in the plastid genome are those under the greatest selection. A combination of both factors may occur. These results for plastid-encoded and nucleus-encoded plastid-targeted proteins are an important indication of the relative distances between the species for which the rates of divergence of the nucleomorph genomes can be compared.

### Nucleomorph encoded, non-plastid proteins

Previous phylogenetic observations of nucleomorph-encoded proteins, have led to speculation that the nucleomorph genomes are extraordinarily divergent, however these studies have been made of proteins that do not target to the plastid. The nucleomorph genomes of *G. theta *and *B. natans *each only encode a handful of plastid proteins, and even fewer for periplastidial metabolism. The rest of the genes encode proteins to support the nucleomorph; proteins for transcription, translation, protein folding and degradation and RNA metabolism [7,8]. These proteins are active within this discrete and reduced cellular space and do not interact with very many other proteins, and therefore selection pressure is hypothesized to be relaxed resulting in proteins of greater relative divergence.

To test this, we selected nucleomorph-encoded genes for proteins that function in the periplastidal space, and compared the rates of evolution of these genes with homologues from nuclear genomes (black, Figure 2a). Average distances between nucleomorph-encoded proteins of *G. theta *and nuclear-encoded homologues from *A. thaliana *&*C. merolae *are larger than the distances between proteins that are plastid-encoded in all species (red), whereas there is less difference between these protein distances and those of proteins that are nucleus-encoded in all species (blue). However, significantly, the relative distances between taxa are not equal. The distance to *G. theta *from both *A. thaliana *and *C. merolae *is greater than the difference between *A. thaliana *and *C. merolae *(black, Figure 2a). This is consistent with relaxed selective pressure for proteins in the periplastidal space. This trend is even more pronounced in the chlorarachniophyte. Average distances between nucleomorph-encoded proteins of *B. natans*, and nuclear-encoded homologues from *A. thaliana *&*C. reinhardtii *(black, Figure 2b) are larger than either plastid (red) or nucleus-encoded plastid-targeted proteins (blue), and the distances are also not equal. The distance to *B. natans *from both *A. thaliana *and *C. reinhardtii *is much greater than the difference between *A. thaliana *and *C. reinhardtii *(black, Figure 2b).

Overall, this confirms expectations that protein-coding genes encoded and active in the nucleomorph and periplastidal space are accumulating mutations faster than nuclear or plastid-encoded proteins. By themselves, however, these observations do not allow us to distinguish between rapid mutation rates in the nucleomorph genomes as opposed to relaxed selective pressures on proteins active within the periplastidal space.

### The rate of divergence of nucleomorph-encoded plastid-targeted proteins is restrained in cryptomonads but not in chlorarachniophytes

The nucleomorph of *G. theta *contains 19 genes that encode plastid-targeted proteins of known function [7]. Of these, only two isoforms of Clp protease, and Cpn60 are also represented in the nucleomorph of *B. natans*, (the other 16 genes are not common to *B. natans*), which contains 14 further genes encoding proteins targeted to the plastid [8].

Why these plastid-targeted proteins remain encoded in the nucleomorph may be the key to the existence of the genome itself, since almost all other nucleomorph-encoded proteins are for self-maintenance and expression of the genome. A variety of biological explanations have been suggested for the retention of certain core proteins in most chloroplast and mitochondrial genomes [51,52], however, given that the nucleomorph is itself a remnant nucleus none of these apply to nucleomorphs. It remains a possibility that, despite there being almost no overlap in plastid-protein content, these proteins are retained in each genome for biological reasons specific to each system, as hypothesized for core genes of the mitochondrial and plastid genomes. Alternatively, they may be genes that simply have not yet been successfully transferred to the nucleus. Indeed, in this study we identified a nuclear copy of a nucleomorph gene, *tha*4, which may have led to the demise of the nucleomorph-encoded gene relatively recently showing the ongoing nature of the process. By extension, it is possible that only the few genes whose proteins are more permissive to mutation can tolerate the high mutation rate of nucleomorph genomes. Selection pressure favouring the successful transfer of genes for proteins under tighter selection for sequence conservation would be stronger. This would suggest that the genes for plastid-targeted proteins remaining in the nucleomorphs would be divergent compared with homologues in other eukaryotes, perhaps as divergent as other nucleomorph proteins on average.

To test these hypotheses, we first compared the relative distances of nucleomorph-encoded plastid-targeted proteins to nucleus-encoded plastid-targeted and plastid-encoded proteins (Figure 2). Fifteen nucleomorph-encoded plastid-targeted proteins of *G. theta *had identifiable homologues in the nuclear genomes of *C. merolae *and *A. thaliana *and 17 nucleomorph-encoded plastid-targeted proteins of *B. natans *had identifiable homologues in the nuclear genomes of *C. reinhardtii *and *A. thaliana*.

Average distances between nucleomorph-encoded plastid-targeted proteins from *G. theta *and nuclear-encoded homologues from *A. thaliana *and *C. merolae *are larger than plastid-encoded proteins, but similar to nucleus-encoded plastid-targeted proteins. The distances between the three species are not equal. As for the plastid-encoded proteins, the distance to *A. thaliana *from both *G. theta *and *C. merolae *is much greater than the difference between *G. theta *and *C. merolae *(green, Figure 2a). Again, this indicates that red and green plastids are more distant than primary and secondary red plastids (as expected). However this result is interesting because it is contrary to the results obtained for nucleomorph-encoded non-plastid proteins, which suggested that nucleomorph proteins were evolving at a faster rate. In the case of the chlorarachniophyte, average distances between nucleomorph-encoded plastid-targeted proteins from *B. natans*, and nucleus-encoded homologues from *A. thaliana *and *C. reinhardtii *are also greater than plastid-encoded proteins. In this case, however, the results contrast sharply with *G. theta *because the distance to *B. natans *from both *A. thaliana *and *C. reinhardtii *is much greater than the difference between *A. thaliana *and *C. reinhardtii *(green, Figure 2b), showing that in this case both types of nucleomorph-encoded proteins (plastid and periplastidal) have experienced accelerated evolution.

Relative rate tests can be used to measure the degree of divergence of two genes from an equally distant outgroup [53,54]. Relative rate tests were performed to determine differences in rates of evolution of individual genes encoding plastid-targeted proteins from the three genomes of both *B. natans *and *G. theta *and their homologues in the green alga *C. reinhardtii *and the red algal *C. merolae*. *A. thaliana *was used as an outgroup for both the *B. natans *and *G. theta *datasets. Relative rates were calculated using RRTree [55] and were tested at a 95% confidence interval (Table 2). Nucleomorph-encoded plastid proteins in *B. natans *fail the relative rate test at a 95% confidence level at a far high frequency than plastid proteins encoded in either the chloroplast or nuclear genomes. Of the plastid proteins encoded in the *B. natans *nucleomorph genome, 82% fail the relative rate test, in each case the peptide is evolving more rapidly in *B. natans*. Similar proportions of nuclear-encoded plastid-targeted proteins (33%) and plastid-encoded proteins (37%) fail the relative rate test in *B. natans *in which cases *B. natans *is typically the most rapidly evolving peptide. In *G. theta*, nucleomorph-encoded plastid-targeted proteins fail the relative rate test more frequently than those encoded in the plastid or nucleus, but the difference is not nearly as pronounced as in *B. natans*. In fact, nucleomorph encoded plastid-targeted proteins in *G. theta *only fail the relative rate test 11% more frequently than nuclear-encoded plastid-targeted proteins in which *G. theta *is the most rapidly evolving taxon. Interestingly, of the 17% of the plastid-encoded peptides that fail the relative rate test, *G. theta *is not the most rapidly evolving ingroup. This may indicate that the plastid of *C. merolae *is evolving at an accelerated rate compared to that of *G. theta*.

**Table 2:** Percentage relative rates rest (calculated by RRTree) failures (P < 0.05; 95% confidence) of plastid proteins encoded in three genomes

**Organism**	**Genome**	**% Failure**	**% failure when the *B. natans *or *G. theta *encoded protein is evolving faster**
*G. theta*	plastid	17	**0**
	nucleus	26	**22**
	nucleomorph	33	**33**
*B. natans*	plastid	37	**31**
	nucleus	33	**33**
	nucleomorph	82	**82**

Overall, the rate of evolution of plastid proteins encoded in the nucleomorph of cryptomonads is in line with those encoded in the nucleus, despite the fact that other nucleomorph-encoded proteins are generally evolving at a higher rate. In chlorarachniophytes, however, the nucleomorph-encoded plastid-targeted proteins are evolving much faster than those encoded in the nucleus (as was also seen for non-plastid nucleomorph-encoded proteins), which provides one of the first indications that the mode of evolution in these two genomes is fundamentally different.

### The proteins retained in nucleomorph genomes are not fast-evolving in other organisms

**Figure 3:**
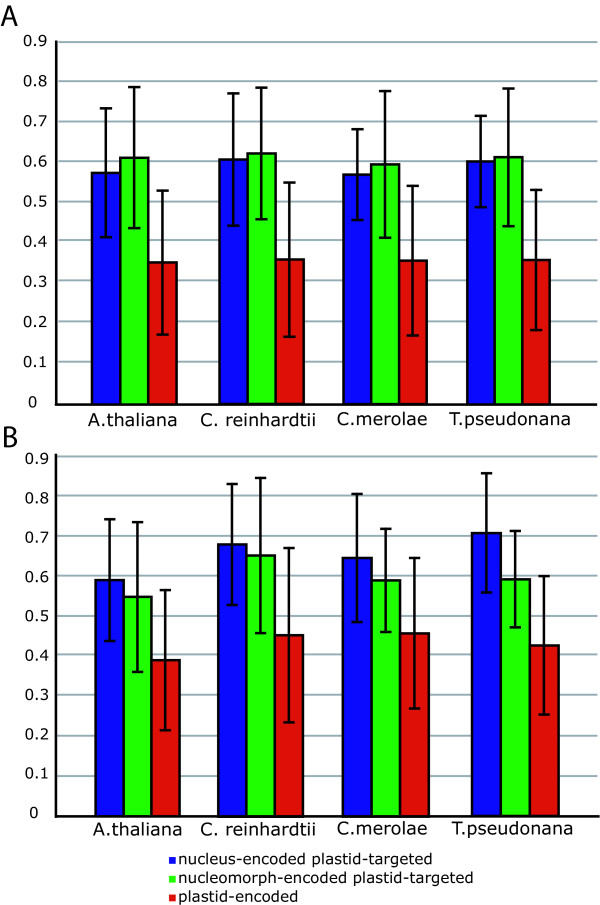
Average distances of homologues from four taxa of plastid proteins encoded in the nucleus (blue), nucleomorph (green) and plastid (red) in (A) *G. theta *and (B) *B. natans *from the cyanobacterium *Synechocystis *sp. PCC 6803.

To further test if the genes retained in the nucleomorph genome are present because the proteins they encode are tolerant of high mutation rates, we compared the evolutionary rates of these proteins in other organisms to the average rates of other plastid-targeted proteins in their nuclear genomes as well as genes retained in the plastid genome. This would reveal if the proteins encoded in the nucleomorph genomes were generally more divergent in all species or not. Since these are proteins of plastid origin, the complete genomes of photosynthetic eukaryotes were used, including the diatom *Thalassiosira pseudonana*, and the distance of these proteins compared to an extant free-living plastid relative; the cyanobacterium *Synechocystis *PCC 6803. This analysis showed that plastid proteins that are encoded in the nucleomorph of either *G. theta *or *B. natans *(green bars, Figure 3) are not significantly more divergent in any other species than plastid-targeted proteins are in general (Figure 3). We should point out that detecting any differences now may be hampered by the fact that all nucleus-encoded plastid-targeted proteins may have existed for some time in a nucleomorph-like genome that has since been lost. This analysis also shows that plastid-encoded proteins are generally less divergent (red bars, Figure 3), as shown in Figure 1, however in this analysis the range of error was large because of the great distance to the cyanobacterium.

### Are cryptomonad nucleomorphs still diverging rapidly?

One of the important observations of prokaryotic enslaved genomes is that, despite the divergence from their free-living relatives, enslaved genomes themselves are generally closely related. For example on a phylogenetic tree of gamma-proteobacteria there is a long branch leading to the *Buchnera aphidicola *clade, but strains of *B. aphidicola *from many aphid species are separated by relatively short distances[56]. This is important because it shows that there are large changes after enslavement, estimated to be 200–250 million years ago [57], but then the genomes become stable [31]. This has been shown in other systems, see table 1. So, these genomes, while highly derived, are apparently stable in this derived condition. In the case of bacterial endosymbionts of invertebrates there is little evidence to suggest that they are becoming organelles and losing genetic information to the host. Similarly, while there may still be some ongoing gene transfer from plastid and mitochondrial genomes [58-61], it seems that a core genome is relatively stable [51,52]. To extrapolate to endosymbiont nuclear genomes, it is critical to know if the rate of divergence between two nucleomorph genomes is similar or different than the rate of divergence between their hosts. If they are behaving as other enslaved genomes do, then the distance will be smaller and perhaps this is one indication of having reached stability. If the forces driving the divergent nature of nucleomorphs are still active, then they will be more divergent than their hosts.

**Figure 4:**
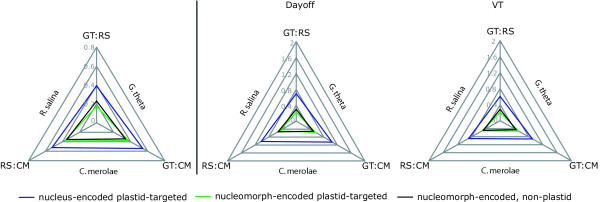
Radar graphs of average distance of nucleus- and nucleomorph-encoded plastid proteins and nucleomorph-encoded non-plastid proteins from the two cryptomonads *R. salina *(RS), *G. theta *(GT) and the free living red algae *C. merolae *(CM).

The average distances between nucleus and nucleomorph-encoded plastid-targeted proteins, and nucleomorph proteins active in the periplastidial space were calculated for two cryptomonads, *G. theta*, and *R. salina*, and compared to their homologues in *C. merolae*. This analysis was made with homologues of six nucleomorph-encoded plastid-targeted protein, six nucleus-encoded plastid-targeted proteins, and nine nucleomorph-encoded non-plastid proteins. The distances between nucleomorph-encoded proteins (both plastid and non-plastid) from *G. theta *and *R. salina *are actually less than the distances between nucleus-encoded proteins (Figure 4). Moreover, for both sets of nucleomorph-encoded proteins and for the nucleus-encoded proteins, the distance to *C. merolae *from both *G. theta *and *R. salina *is greater than the distance between *G. theta *and *R. salina*. The distance between *R. salina *and *G. theta *for nucleomorph non-plastidproteins is slightly greater than for plastid-targeted proteins. Taken together, these results suggest that the nucleomorph proteins of cryptomonads are not diverging rapidly but, like their plastid genomes, are evolving at a slower rate than their nuclear genomes. However, the proteins not targeted to the plastid are slightly less constrained than those proteins targeted to the plastid.

## Conclusion

Our analyses show that nucleus-encoded plastid-targeted proteins are, on average, more divergent than proteins encoded in the plastid genome. Although the results cannot explain the reason for this difference, because the proteins encoded in both genomes are active in the same cellular compartment, the plastid, we assume that they are under similar selection pressures and so the difference is more likely to be attributed to a higher rate of substitution in the nuclear genome than to differences in selection pressure. Similarly we confirmed the expectations that protein-coding genes encoded and active in the nucleomorph have accumulated more mutations than nuclear or plastid-encoded proteins but again cannot distinguish between rapid mutation rates in the nucleomorph genomes as opposed to relaxed selective pressures on proteins active within the periplastidal space.

Two more significant results, however, come from the nucleomorph genomes. First, nucleomorph-encoded plastid-proteins reveal differences in the evolution of cryptomonad and chlorarachniophyte nucleomorphs. In *G. theta*, the nucleomorph-encoded plastid-proteins are evolving, on average, at about the same rate as nuclear-encoded plastid proteins. In contrast, *B. natans *nucleomorph-encoded plastid-targeted proteins are evolving much faster than those encoded in the nucleus, and indeed evolve at about the same rate as other nucleomorph proteins. Second, the nucleomorphs of two cryptomonads are diverging less rapidly than their nuclear genomes. The nucleomorph-encoded proteins active in the periplastidial space are somewhat more divergent than plastid-targeted proteins, but still less than nuclear proteins and this may reflect relaxed selection pressure in this compartment. Together with evidence from Lane et al [62], which shows that cryptomonad nucleomorph genomes differ in size but have conserved other properties such as gene order, our results suggest that the nucleomorph genomes of cryptomonad species are not rapidly evolving and are likely relatively conserved. This is comparable to other enslaved genomes such as bacterial endosymbionts and many plastid and mitochondrial genomes. Unfortunately, there is no data from other species of chlorarachniophytes with which to make a similar comparison. From this single species it is difficult to determine whether the nucleomorph genome is stable or not, but by comparison to cryptomonads it seems that the nucleomorph-encoded proteins in *B. natans *are more weakly constrained. It is possible that differences exist between the biology of these two compartments that promote a higher degree of sequence conservation in one lineage than in the other. Just what the underlying causes of such different rates of evolution may be is not obvious, given what is currently known about nucleomorphs, but further information from a greater diversity of chlorarachniophyte nucleomorphs may resolve whether the nucleomorph of *B. natans *is itself evolving rapidly, or whether the ancestor of chlorarachniophyte nucleomorphs underwent a rapid burst of sequence evolution subsequent to the endosymbiotic event that gave rise to the chlorarachniophyte endosymbiont.

## Methods

### Identification of plastid-proteins

Proteins representing known plastid functions from other eukaryotes and cyanobacteria, were used to search ongoing EST projects from the cryptomonads *Guillardia theta *(CCMP 327) and *Rhodomonas salina *(CCMP 1319) and also previously published data from *B. natans *[16,47], resulting in a set of putative nucleus-encoded plastid-targeted protein genes. In the cases of *B. natans *where several lateral gene transfers have been identified [16], only nuclear encoded plastid proteins of chlorophyte origin were used. ESTs were completely sequenced on both strands from over-lapping cDNA clones for each cluster. New sequences analysed here have been deposited in GenBank under accession numbers DQ383756-DQ383799. Proteins were also identified from the coding sequences of the ongoing sequencing project of the plastid genomes of *Bigelowiella natans *and the plastid genomes of *G. theta *[46], *Arabidopsis thaliana *[63], *Cyanidioschyzon merolae *[64] and *Odontella sinesis *[65]. Homologues of plastid-proteins were identified from the nuclear genomes of *Thalassiosira pseudonana *[66], *A. thaliana *[67], *C. merolae *[40]. Proteins sequences were also used from the complete genome of the cyanobacterium *Synechocystis *sp. PCC 6803, and the nucleomorph genomes of *G. theta *[7] and *B. natans *(DQ158856 – DQ158858). When multiple isoforms existed in the algal or plant nucleus and it was not obvious which isoform was the orthologue, the distances for all isoforms were calculated and the isoforms with the closest distance to the cryptomonad or chlorarachniophyte was used, providing that the same isoforms from the algae and plant were also closest to each other. Alternatively, in a few cases, a neighbour-joining phylogenetic tree was constructed to determine groups of isoforms. In a minority of cases for nucleomorph-encoded plastid proteins in *B. natans *where there were multiple paralogues in both *A. thaliana *and *C. reinhardtii*, the nearest *Arabidopsis *paralogue to *B. natans *was not nearest to the *C. reinhardtii *paralogue closest to *B. natans*. In these cases the paralogue closest to *B. natans *in pair-wise distance (using Dayhoff) was chosen. If it was not possible to determine which isoform was the likely original paralogue then that protein was excluded from the analysis. For analyses with nucleomorph-encoded non-plastid proteins a subset of proteins involved in transcription, translation (ribosomal subunits excepted) and protein folding for which homologues could be identified in *A. thaliana *and *C. reinhardtii *or *C. merolae*, was used.

### Identification of *R. salina *nucleomorph transcripts

Proteins encoded in the nucleomorph genome of *Guillardia theta *were used to search a database of *Rhodomonas salina *(CCMP 1319) ESTs using tBLASTn. The GC content of the transcripts was calculated and compared to the GC content of the *G. theta *nucleomorph and nuclear genome and also to *R. salina *proteins identified as being nuclear-encoded, plastid-targeted. *R. salina *transcripts with homologues in the *G. theta *nucleomorph with coding regions of 28% GC content or less were determined to be nucleomorph encoded.

### Calculation of distances

Protein alignments were made using Clustal X [68] and refined in MacClade (Sinauer Associates, MA. USA). Distances were calculated using PAUP 4.0b10 (Sinauer Associates, MA. USA) and TREE-PUZZLE 5.2 [69] with either the Dayhoff or VT substitution matrix. For comparisons to *G. theta *distances were also calculated with the Dayhoff substutution matrix and nine rates catagories (eight variable and one invariable), to test for saturation [see Additional file 1].

### Relative rates

Relative rate tests were performed using the RRTREE program [55] using *C. reinhardtii *as an ingroup and *A. thaliana *as an outgroup for *B. natans *datasets. *C. merolae *was used as an ingroup and *A. thaliana *as an outgroup for *G. theta *datasets. The test was used to compare the evolutionary rate of individual genes from each of the three genomes of *B. natans *and *G. theta *to its compartment specific homologue in the genomes of *C. reinhardtii *and *C. merolae*. Since a failure of a relative rate test does not indicate which taxon is evolving more rapidly, we compare failures where *G. theta *or *B. natans *is the most rapidly evolving ingroup.

## Abbreviations

EST, Expressed Sequence Tag

## Authors' contributions

All authors contributed to the experimental concept and design, the generation of EST data, data acquisition, analysis, statistics, and manuscript preparation. All authors read and approved the final manuscript.

## Supplementary Material

Additional File 1 Supplementary Figure 1. Radar graph of average distances of plastid-, nucleus-, and nucleomorph-encoded plastid proteins, and nucleomorph-encoded non-plastid proteins of the cryptophyte *G. theta *(GT), the red algae *C. merolae *(CM) and the plant *A. thaliana *(AT) calculated with the Dayhoff substitution matrix and nine catagories of gamma corrections (eight variable +1 invariable). Click here for file

## References

[B1] Gray MW, Spencer DF, Roberts DM, Sharp P, Alderson  G and Collins MA (1996). Organellar Evolution. Evolution of Microbial Life.

[B2] Cavalier-Smith T (2003). Genomic reduction and evolution of novel genetic membranes and protein-targeting machinery in eukaryote-eukaryote chimaeras (meta-algae). Philos Trans R Soc Lond B Biol Sci.

[B3] Archibald JM, Keeling PJ (2002). Recycled plastids: a 'green movement' in eukaryotic evolution. Trends Genet.

[B4] Martin W, Herrmann RG (1998). Gene transfer from organelles to the nucleus: How much, what happens, and why?. Plant Physiology.

[B5] Archibald JM, Keeling PJ (2003). Comparative genomics. Plant genomes: cyanobacterial genes revealed. Heredity.

[B6] Nozaki H, Matsuzaki M, Misumi O, Kuroiwa H, Hasegawa M, Higashiyama T, Shin T, Kohara Y, Ogasawara N, Kuroiwa T (2004). Cyanobacterial genes transmitted to the nucleus before divergence of red algae in the chromista. Journal of Molecular Evolution.

[B7] Douglas S, Zauner S, Fraunholz M, Beaton M, Penny S, Deng LT, Wu X, Reith M, Cavalier-Smith T, Maier UG (2001). The highly reduced genome of an enslaved algal nucleus. Nature.

[B8] Gilson P, Mollard V, Slamovits CH, Reith M, Keeling PJ, McFadden G (2006). Complete nucleotide sequence of the chlorarachniophyte nucleomorph. Proc Natl Acad Sci U S A.

[B9] Gilson PR, McFadden GI (1997). Good things in small packages: the tiny genomes of chlorarachniophyte endosymbionts. Bioessays.

[B10] Gilson PR, McFadden GI (2002). Jam packed genomes--a preliminary, comparative analysis of nucleomorphs. Genetica.

[B11] Gould SV, Sommer MS, Hadfi K, Zauner S, Kroth P, Maier UG (2006). Protein Targeting into the Complex Plastid of Cryptophytes. Journal of Molecular Evolution.

[B12] Keeling PJ, Archibald JM, Fast NM, Palmer JD (2004). Comment on "The evolution of modern eukaryotic phytoplankton". Science.

[B13] Keeling P (2004). A brief history of plastids and their hosts. Protist.

[B14] Falkowski PG, Katz ME, Knoll AH, Quigg A, Raven JA, Schofield O, Taylor FJ (2004). The evolution of modern eukaryotic phytoplankton. Science.

[B15] Sulli C, Fang Z, Muchhal U, Schwartzbach SD (1999). Topology of Euglena chloroplast protein precursors within endoplasmic reticulum to Golgi to chloroplast transport vesicles. J Biol Chem.

[B16] Archibald JM, Rogers MB, Toop M, Ishida K, Keeling PJ (2003). Lateral gene transfer and the evolution of plastid-targeted proteins in the secondary plastid-containing alga Bigelowiella natans. Proceedings of the National Academy of Sciences of the United States of America.

[B17] Fast NM, Kissinger JC, Roos DS, Keeling PJ (2001). Nuclear-encoded, plastid-targeted genes suggest a single common origin for apicomplexan and dinoflagellate plastids. Molecular Biology and Evolution.

[B18] Harper JT, Keeling PJ (2003). Nucleus-encoded, plastid-targeted glyceraldehyde-3-phosphate dehydrogenase (GAPDH) indicates a single origin for chromalveolate plastids. Mol Biol Evol.

[B19] Patron NJ, Rogers MB, Keeling PJ (2004). Gene replacement of fructose-1,6-bisphosphate aldolase supports the hypothesis of a single photosynthetic ancestor of chromalveolates. Eukaryotic Cell.

[B20] Cavalier-Smith T (1998). A revised six-kingdom system of life. Biological Reviews.

[B21] Katinka MD, Duprat S, Cornillot E, Metenier G, Thomarat F, Prensier G, Barbe V, Peyretaillade E, Brottier P, Wincker P, Delbac F, El Alaoui H, Peyret P, Saurin W, Gouy M, Weissenbach J, Vivares CP (2001). Genome sequence and gene compaction of the eukaryote parasite Encephalitozoon cuniculi. Nature.

[B22] Williams BA, Slamovits CH, Patron NJ, Fast NM, Keeling PJ (2005). A high frequency of overlapping gene expression in compacted eukaryotic genomes. Proc Natl Acad Sci U S A.

[B23] Foster J, Ganatra M, Kamal I, Ware J, Makarova K, Ivanova N, Bhattacharyya A, Kapatral V, Kumar S, Posfai J, Vincze T, Ingram J, Moran L, Lapidus A, Omelchenko M, Kyrpides N, Ghedin E, Wang S, Goltsman E, Joukov V, Ostrovskaya O, Tsukerman K, Mazur M, Comb D, Koonin E, Slatko B (2005). The Wolbachia genome of Brugia malayi: endosymbiont evolution within a human pathogenic nematode. PLoS Biol.

[B24] Pfarr K, Hoerauf A (2005). The annotated genome of Wolbachia from the filarial nematode Brugia malayi: what it means for progress in antifilarial medicine. PLoS Med.

[B25] (2004). Genome Sequence of the Intracellular Bacterium Wolbachia. PLoS Biol.

[B26] Shigenobu S, Watanabe H, Hattori M, Sakaki Y, Ishikawa H (2000). Genome sequence of the endocellular bacterial symbiont of aphids Buchnera sp. APS. Nature.

[B27] Akman L, Yamashita A, Watanabe H, Oshima K, Shiba T, Hattori M, Aksoy S (2002). Genome sequence of the endocellular obligate symbiont of tsetse flies, Wigglesworthia glossinidia. Nat Genet.

[B28] Wernegreen JJ, Lazarus AB, Degnan PH (2002). Small genome of Candidatus Blochmannia, the bacterial endosymbiont of Camponotus, implies irreversible specialization to an intracellular lifestyle. Microbiology.

[B29] Ochman H (2005). Genomes on the shrink. Proceedings of the National Academy of Sciences of the United States of America.

[B30] Wernegreen JJ (2004). Endosymbiosis: Lessons in conflict resolution. Plos Biology.

[B31] Silva FJ, Latorre A, Moya A (2003). Why are the genomes of endosymbiotic bacteria so stable?. Trends in Genetics.

[B32] Schaber J, Rispe C, Wernegreen J, Buness A, Delmotte F, Silva FJ, Moya A (2005). Gene expression levels influence amino acid usage and evolutionary rates in endosymbiotic bacteria. Gene.

[B33] Rispe C, Delmotte F, van Ham R, Moya A (2004). Mutational and selective pressures on codon and amino acid usage in Buchnera, endosymbiotic bacteria of aphids. Genome Research.

[B34] Wernegreen JJ, Moran NA (1999). Evidence for genetic drift in endosymbionts (Buchnera): Analyses of protein-coding genes. Molecular Biology and Evolution.

[B35] Birdsell JA (2002). Integrating genomics, bioinformatics, and classical genetics to study the effects of recombination on genome evolution. Molecular Biology and Evolution.

[B36] Wilson RJ, Denny PW, Preiser PR, Rangachari K, Roberts K, Roy A, Whyte A, Strath M, Moore DJ, Moore PW, Williamson DH (1996). Complete gene map of the plastid-like DNA of the malaria parasite Plasmodium falciparum. J Mol Biol.

[B37] Gardner MJ, Hall N, Fung E, White O, Berriman M, Hyman RW, Carlton JM, Pain A, Nelson KE, Bowman S, Paulsen IT, James K, Eisen JA, Rutherford K, Salzberg SL, Craig A, Kyes S, Chan MS, Nene V, Shallom SJ, Suh B, Peterson J, Angiuoli S, Pertea M, Allen J, Selengut J, Haft D, Mather MW, Vaidya AB, Martin DM, Fairlamb AH, Fraunholz MJ, Roos DS, Ralph SA, McFadden GI, Cummings LM, Subramanian GM, Mungall C, Venter JC, Carucci DJ, Hoffman SL, Newbold C, Davis RW, Fraser CM, Barrell B (2002). Genome sequence of the human malaria parasite Plasmodium falciparum. Nature.

[B38] Chanda I, Pan A, Dutta C (2005). Proteome composition in Plasmodium falciparum: Higher usage of GC-rich nonsynonymous codons in highly expressed genes. Journal of Molecular Evolution.

[B39] Peixoto L, Fernandez V, Musto H (2004). The effect of expression levels on codon usage in Plasmodium falciparum. Parasitology.

[B40] Matsuzaki M, Misumi O, Shin-I T, Maruyama S, Takahara M, Miyagishima SY, Mori T, Nishida K, Yagisawa F, Yoshida Y, Nishimura Y, Nakao S, Kobayashi T, Momoyama Y, Higashiyama T, Minoda A, Sano M, Nomoto H, Oishi K, Hayashi H, Ohta F, Nishizaka S, Haga S, Miura S, Morishita T, Kabeya Y, Terasawa K, Suzuki Y, Ishii Y, Asakawa S, Takano H, Ohta N, Kuroiwa H, Tanaka K, Shimizu N, Sugano S, Sato N, Nozaki H, Ogasawara N, Kohara Y, Kuroiwa T (2004). Genome sequence of the ultrasmall unicellular red alga Cyanidioschyzon merolae 10D. Nature.

[B41] The Chlamydomonas reinhartdii genome sequencing project. http://genome.jgi-psf.org/Chlre3/Chlre3.home.html.

[B42] CavalierSmith T, Couch JA, Thorsteinsen KE, Gilson P, Deane JA, Hill DRA, McFadden GI (1996). Cryptomonad nuclear and nucleomorph 18S rRNA phylogeny. European Journal of Phycology.

[B43] Archibald JM, Cavalier-Smith T, Maier U, Douglas S (2001). Molecular chaperones encoded by a reduced nucleus: the cryptomonad nucleomorph. J Mol Evol.

[B44] Keeling PJ, Deane JA, Hink-Schauer C, Douglas SE, Maier UG, McFadden GI (1999). The secondary endosymbiont of the cryptomonad Guillardia theta contains alpha-, beta-, and gamma-tubulin genes. Mol Biol Evol.

[B45] VanDePeer Y, Rensing SA, Maier UG, DeWachter R (1996). Substitution rate calibration of small subunit ribosomal RNA identifies chlorarachniophyte endosymbionts as remnants of green algae. Proceedings of the National Academy of Sciences of the United States of America.

[B46] Douglas SE, Penny SL (1999). The plastid genome of the cryptophyte alga, Guillardia theta: complete sequence and conserved synteny groups confirm its common ancestry with red algae. J Mol Evol.

[B47] Rogers MB, Archibald JM, Field MA, Li C, Striepen B, Keeling PJ (2004). Plastid-targeting peptides from the chlorarachniophyte Bigelowiella natans. Journal of Eukaryotic Microbiology.

[B48] McFadden GI (1999). Plastids and protein targeting. Journal of Eukaryotic Microbiology.

[B49] Patron NJ, Waller RF, Archibald JM, Keeling PJ (2005). Complex protein targeting to dinoflagellate plastids. J Mol Biol.

[B50] Patron NJ, Keeling PJ (2005). Common evolutionary origin of starch biosynthetic enzymes in green and red algae. Journal of Phycology.

[B51] Race HL, Herrmann RG, Martin W (1999). Why have organelles retained genomes?. Trends in Genetics.

[B52] Daley DO, Whelan J (2005). Why genes persist in organelle genomes. Genome Biology.

[B53] Sarich VM, Wilson AC (1973). Generation time and genomic evolution in primates. Science.

[B54] Wu CI, Li WH (1985). Evidence for higher rates of nucleotide substitution in rodents than in man. Proc Natl Acad Sci U S A.

[B55] Robinson-Rechavi M, Huchon D (2000). RRTree: Relative-Rate Tests between groups of sequences on a phylogenetic tree. Bioinformatics.

[B56] Herbeck JT, Degnan PH, Wernegreen JJ (2005). Nonhomogeneous model of sequence evolution indicates independent origins of primary endosymbionts within the enterobacteriales (gamma-Proteobacteria). Mol Biol Evol.

[B57] Baumann P, Baumann L, Lai CY, Roubakhsh D, Moran NA, Clark MA (1995). Genetics, Physiology, and Evolutionary Relationships of the Genus Buchnera - Intracellular Symbionts of Aphids. Annual Review of Microbiology.

[B58] Ayliffe MA, Scott NS, Timmis JN (1998). Analysis of plastid DNA-like sequences within the nuclear genomes of higher plants. Molecular Biology and Evolution.

[B59] Noutsos C, Richly E, Leister D (2005). Generation and evolutionary fate of insertions of organelle DNA in the nuclear genomes of flowering plants. Genome Research.

[B60] Richly E, Leister D (2004). NUPTs in sequenced eukaryotes and their genomic organization in relation to NUMTs. Molecular Biology and Evolution.

[B61] Richly E, Leister D (2004). NUMTs in sequenced eukaryotic genomes. Molecular Biology and Evolution.

[B62] Lane CE, Khan H, Mackinnon M, Fong A, Theophilou S, Archibald JM (2005). Insight into the Diversity and Evolution of the Cryptomonad Nucleomorph Genome. Mol Biol Evol.

[B63] Sato S, Nakamura Y, Kaneko T, Asamizu E, Tabata S (1999). Complete structure of the chloroplast genome of Arabidopsis thaliana. DNA Res.

[B64] Ohta N, Matsuzaki M, Misumi O, Miyagishima SY, Nozaki H, Tanaka K, Shin IT, Kohara Y, Kuroiwa T (2003). Complete sequence and analysis of the plastid genome of the unicellular red alga Cyanidioschyzon merolae. DNA Res.

[B65] Kowallik KV, Stoebe B, Schaffran I, KrothPancic P, Freier U (1995). The chloroplast genome of a chlorophyll a+c-containing alga, Odontella sinensis. Plant Molecular Biology Reporter.

[B66] Armbrust EV, Berges JA, Bowler C, Green BR, Martinez D, Putnam NH, Zhou SG, Allen AE, Apt KE, Bechner M, Brzezinski MA, Chaal BK, Chiovitti A, Davis AK, Demarest MS, Detter JC, Glavina T, Goodstein D, Hadi MZ, Hellsten U, Hildebrand M, Jenkins BD, Jurka J, Kapitonov VV, Kroger N, Lau WWY, Lane TW, Larimer FW, Lippmeier JC, Lucas S, Medina M, Montsant A, Obornik M, Parker MS, Palenik B, Pazour GJ, Richardson PM, Rynearson TA, Saito MA, Schwartz DC, Thamatrakoln K, Valentin K, Vardi A, Wilkerson FP, Rokhsar DS (2004). The genome of the diatom Thalassiosira pseudonana: Ecology, evolution, and metabolism. Science.

[B67] (2000). Analysis of the genome sequence of the flowering plant Arabidopsis thaliana. Nature.

[B68] Thompson JD, Gibson TJ, Plewniak F, Jeanmougin F, Higgins DG (1997). The CLUSTAL_X windows interface: flexible strategies for multiple sequence alignment aided by quality analysis tools. Nucleic Acids Research.

[B69] Schmidt HA, Hasseler A, Baxevanis DB, RDM D, G P, G S and L S (2003). Maximum-Likelihood Analysis Using TREE_PUZZLE. Current Protocols in Bioinformatics.

